# Development of the Management of Out-of-hospital Cardiac Arrest Patients in Japan

**DOI:** 10.31662/jmaj.2020-0114

**Published:** 2021-01-14

**Authors:** Mitsuaki Isobe

**Affiliations:** 1 Sakakibara Heart Institute

**Keywords:** resuscitation, out-of-hospital cardiac arrest, automated external defibrillator, COVID-19

This paper written by Nakahara et al.^(1)^ is a review article describing the history of the development of the legal and environmental conditions of emergency resuscitation and improvements in the survival of out-of-hospital cardiac arrest (OHCA) patients in Japan. This is a precious record of how the laborious and persistent efforts of the persons concerned have brought about progress in science, amendments of laws, and improved awareness of the general public and how the present marked improvements have been realized in the resuscitation rate in OHCA patients. As mentioned by the authors, resuscitation in Japan, which has long trailed Western countries, has at last caught up with the Western level via several legal reforms and public education activities by academic society and civilian bodies. As the paper highlights, Utstein-style all-case survey has been identified to play a vital role. Data collected using an internationally standardized format have made global assessment possible, and, in Japan, scientific validation based on big data, annually reaching a world-leading number of 100,000 cases, has been started.

In 2018, 127,718 OHCA patients have been recorded in Japan, and the number has seen an increasing trend, which could be attributed to its aging population. At the same time, the number of those who attend emergency care training seminars has also increased, exceeding 1.5 million. In association, the resuscitation and rehabilitation rates of OHCA patients have continued to increase. Simultaneously, the rate of OHCA patients given emergency treatment by lay citizens has also risen significantly, and, of the OHCA patients urgently transported in 2018, the 1-month survival rate was 17.5%, and the rate of reintegration into society was 12.5%; in the 25,756 patients witnessed by citizens to have suffered cardiogenic OHCA, the reintegration rate of those who received cardiopulmonary resuscitation (CPR) by bystanders was 48.2%, all marking the highest figures ever^(2)^. These could be attributed to the excellent performance of emergency life-saving technicians and life-saving efforts by bystanders based on the development of legal conditions and improved knowledge of the general public. The sustained efforts by related persons during this period must be appreciated.

As observed by Nakahara et al., the availability of automated external defibrillators (AEDs) has markedly increased during the same period. AEDs are now placed in several sites not only in public spaces, such as amusement centers, railway stations, and sports facilities, but also in private firms and schools. This has also contributed greatly to the improvements in the resuscitation and rehabilitation rates of OHCA patients. In 2016, more than 800,000 units of AED were sold, and about 600,000 are considered to have been installed, which can be considered a steady increase. The increase in basic life support training sessions sponsored by various organizations is also a result of the awareness-raising activities conducted during this period.

In 2018, the nationwide action against cardiovascular diseases in Japan was established^(3)^, and the Basic Plan for Promotion of Countermeasures for Cardiovascular Disease^(4)^ was declared by the government in 2020. This plan also covers the reform of the emergency care system for cardiovascular diseases. Presently, primary to tertiary emergency care hospitals are designated, and each local government has a system for emergency transport of patients. However, the transportation system is changing markedly with the development of highways and increased availability of helicopter transport. Since medical facilities that are able to provide advanced care for ischemic heart diseases and aortic emergencies leading to OHCA are limited, and since the population, age structure, and distribution of medical organizations are markedly changing, the development of a transport system that can flexibly respond to acute cardiovascular accidents has been anticipated. The Basic Plan for Promotion of Countermeasures for Cardiovascular Disease urges continuous review of the standards for the implementation of patient transport and reception suited for the local circumstances and disease condition. In terms of the rescue crew, enhancement of the medical control system for management of patients and improvements in knowledge and skill based on scientific data should be developed under the Basic Plan. With regard to medical control, further advances in online control using new communication tools and remote medical care devices are anticipated, and, for this, appropriate legal guarantee is considered necessary.

Although efforts are being made, many challenges still remain. The importance of bystander CPR must be emphasized more. Further propagation of AEDs, public awareness, and skill of CPR are still important matters that need further consideration. It is also desirable to establish a post hoc assessment system using AED data. Japan will be expected to deliver scientific evidence using its world’s largest database and perform assessment by high-quality RCTs.

The COVID-19 pandemic, which emerged in 2020, has been significantly impacting the approaches to OHCA. In New York, OHCA has reportedly increased tenfold during the pandemic^(5)^. Although the Japan Resuscitation Council has already made proposals in some of its manuals, evidence remains lacking, and confusion of the clinical front line is expected to persist. Thus, evaluation and review of the appropriateness of the approaches taken in this situation including the methods of enforcement of the training course and indications and methods of CPR are necessary.

As the authors wish, I hope that this review paper will contribute to improvements in the resuscitation rate in countries and regions where the life-saving system for OHCA must still be developed.

## Article Information

### Conflicts of Interest

None

### Disclaimer

Mitsuaki Isobe is one of the Associate Editors of JMA Journal and on the journal’s Editorial Staff. He was not involved in the editorial evaluation or decision to accept this article for publication at all.
